# Case report: primary resistance to osimertinib in erlotinib-pretreated lung adenocarcinoma with EGFR T790 M mutation

**DOI:** 10.1186/s12885-018-4991-4

**Published:** 2018-11-06

**Authors:** Lin-Peng Zheng, Li-Ying Chen, Xing-Yun Liao, Zi-Han Xu, Zheng-Tang Chen, Jian-Guo Sun

**Affiliations:** Department of Oncology, Xinqiao Hospital, Army Medical University, Chongqing, 400037 China

## Abstract

**Background:**

Among non-small cell lung cancer (NSCLC) patients with acquired T790 M mutation resistance to first-generation epidermal growth factor receptor-tyrosine kinase inhibitor (EGFR-TKI), 71% are likely to benefit from osimertinib. There have been several reports about the secondary resistance to osimertinib treatment in T790 M-positive patients, while primary resistance to osimertinib has been rarely reported.

**Case presentation:**

A 62-year-old Asian male never smoker who presented with stage IV EGFR L858R-positive adenocarcinoma developed EGFR T790 M mutation after 14 months of treatment with erlotinib combined with thoracic radiotherapy as first-line therapy. The patient was initiated on osimertinib treatment with T790 M mutation detected (14.4%), but disease progressed 2 months later.

**Conclusion:**

The mechanism of primary resistance to osimertinib remains unclear. There may be an association between T790 M mutation disappearance, TP53 mutation and radiotherapy, but further researches are needed to confirm this.

## Backgound

AURA3 study showed that the patients who failed in the first-generation EGFR-TKI therapy acquired 10.1 months of median PFS (mPFS) after taking osimertinib [1]. However, some of them may also resist to osimertinib after a few months, which was termed secondary resistance. To our knowledge, there have been rare reports about primary resistance to osimertinib. Herein, we report a case of primary resistance to osimertinib.

## Case description

**Fig. 1:**
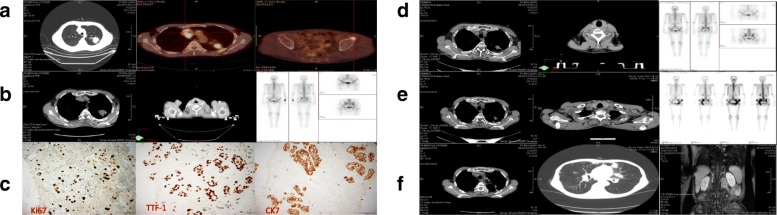
diagnosis (cT2N3M1b) and tumor response in the first-, second-, third-, and fourth-line treatments. The first-line treatment (erlotinib plus radiotherapy, December 2014) (**a**/**b**) CT scans showed a mass (3.8 cm* 3.3 cm) on the left upper lobe on December 29, 2014; PET/CT found the mass and multiple enlarged lymph nodes at bilateral neck, clavicle, left pulmonary portal, mediastinum, the 4th thoracic vertebra and left acetabulum, and showed left sciatic metastasis; Whole-body bone scintigraphy showed abnormal metabolism of the anterior superior iliac spine; (**c**) Immunohistochemistry staining showed high expression of CK7, TTF-1 and Ki67. Original magnification × 200. **d** The second-line treatment (local radiotherapy, March 2016). Thoracic CT and Whole-body bone scintigraphy showed the lung mass and bone metastases were stable, but bilateral neck and right supraclavicular lymph nodes were slightly larger than before. **e** The third-line treatment (osimertinib plus local radiotherapy, April 2017). Whole-body bone scintigraphy found more bone metastases. Thoracic CT showed the lung mass was stable but cervical lymph nodes reappeared. **f** The fourth-line treatment (apatinib, August 2017). Thoracic CT showed the mass and nodules (especially lesion in the upper lobe of the left lung) were bigger. Lumbar MRI showed a mass of 5.5 cm *2.9 cm in the left appendage area of the 1st and 2nd lumbar

A 62-year-old male never smoker presented with several painless but slowly enlarging lymph nodes in the bilateral neck in December 2014. After a series of examinations (Fig. 1a-c), the patient was diagnosed with lung adenocarcinoma of the left upper lobe (stage IV, cT2N3M1b) harboring L858R mutation in exon 21 of EGFR gene in January, 2015.

The patient was recruited to a clinical trial (NCT 02353741) and administered with erlotinib (150 mg/d) plus radiotherapy in left lung and mediastinum (PGTV60Gy/30F/6W) from January 8, 2015. Partial response (PR) was identified in this patient according to the Response Evaluation Criteria in Solid Tumors (RECIST) (version 1.1).

Disease progressed in March 2016. Neck CT found enlarged right supraclavicular nodules and axillary lymph nodes (Fig. 1d). Resection biopsy of the right supraclavicular lymph node found EGFR T790 M mutation in exon 20 (detected by ARMS-qPCR), but the lung lesions did not change much (Fig. 1d). Therefore, local radiotherapy was adopted. After following up from April 7, 2016 to January 4, 2017, the tumor response was assessed and stable disease (SD) was achieved.

Pelvis magnetic resonance imaging (MRI) and whole-body bone scintigraphy (Fig. 1e) showed multiple bone metastases in April 2017. Resection biopsy of supraclavicular lymph node revealed that there was no pathological transformation. Peripheral blood molecular detection found EGFR T790 M mutation (14.4%). Thus, the patient received second-line treatment with oral osimertinib (80 mg/day) combined with radiotherapy of bilateral ischia (PGTV 54Gy/18F). No other systemic therapy was added.

**Fig. 2:**
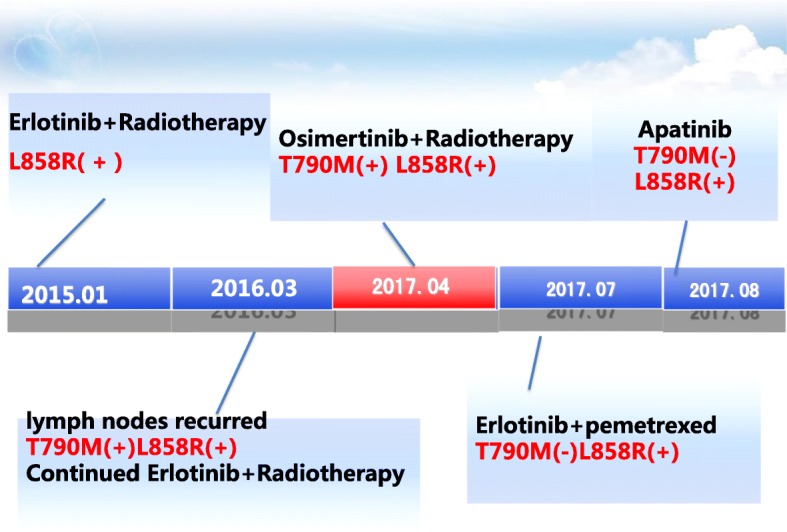
Treatment history

However, thoracic CT identified pulmonary nodule progression (progressive disease, PD) two months later, and the patient’s performance status (PS) didn’t improve. Resection biopsy of the left axillary lymph node showed that EGFR L858R mutation still existed, but T790 M mutation disappeared. Erlotinib combination with pemetrexed for two cycles from July 4, 2017. A mass of 5.5 cm *2.9 cm growing from the left paravertebral soft tissues of L1–2 and enlarged retroperitoneal lymph nodes in the pelvis were found on August 21, 2017 (Fig. 1f). Core needle biopsy of paravertebral mass revealed no pathological transformation of SCLC (CK +, TTF-1 +, LCA -, Ki-67 50%+). EGFR T790 M mutation was still negative and L858R was positive. The patient was switched to apatinib, a VEGFR2 inhibitor, from August 29, 2017. However, a large amount of pleural effusion was found on September 7, 2017, and PS was 4. One month later, the patient died. A brief introduction to the treatment history was shown in Fig. 2.

## Discussion

**Fig. 3:**
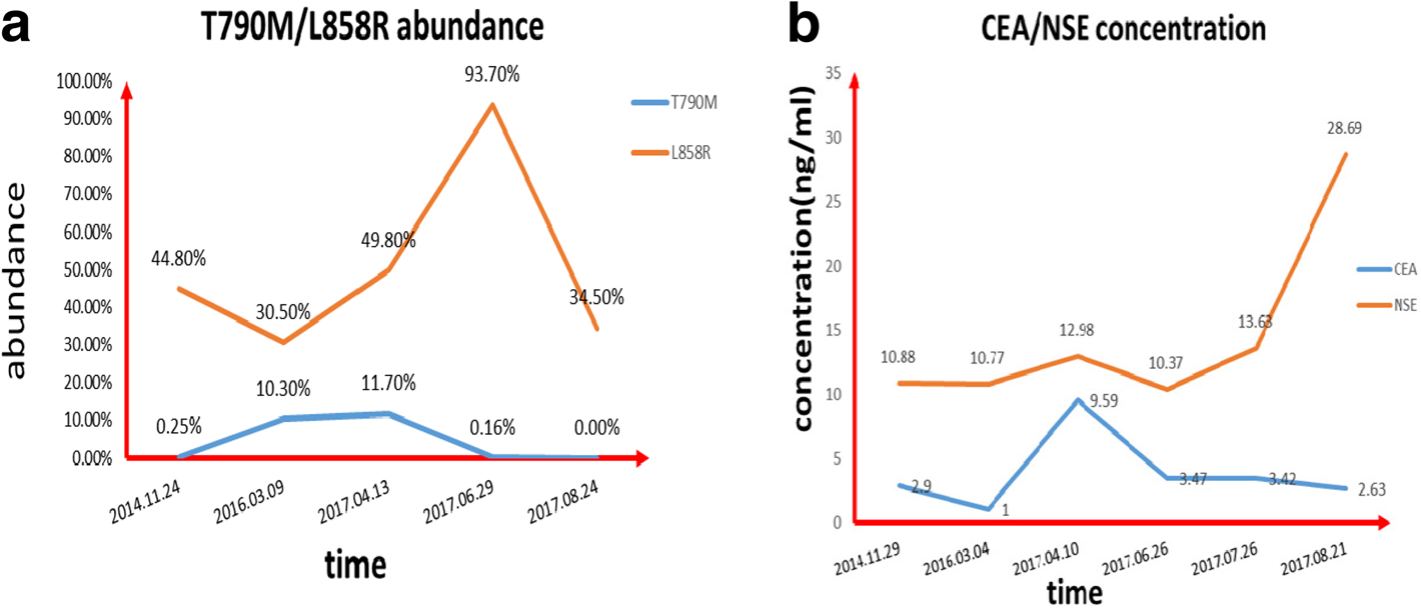
Molecular and pathological analysis. **a** Droplet digital polymerase chain reaction (ddPCR) for retrospective detection of the EGFR L858R and T790 M mutation abundance (tumor tissue). T790 M mutation abundance was 0.25% when the patient was diagnosed, 11.7% when he began to take osimertinib, 0.16% two months after taking osimertinib, and 0% four months after taking osimertinib. **b** Neuron-specific enolase (NSE) level was progressively increasing. CEA level reached peak in June 2017

The mechanism of acquired resistance to osimertinib includes C797S mutation, T790 M disappearance, EGFR amplification, bypass pathway activation, HER2 and MET amplification, phenotypic alterations and so on [2]. Few cases of primary resistance to osimertinib forT790 M mutation have been reported, so we present one case here. In this case, T790 M mutation was positive at first, but became negative after osimertinib treatment (Fig. 3a). So T790 M mutation disappearance could be a possible mechanism of osimertinib resistance. R. Minari [3] reported that SCLC transformation might be the cause of primary resistance to osimertinib. In this case, neuron-specific enolase (NSE) level of the patient was progressively increasing, and the CEA level was slowed down after EGFR-T790 M became negative (Fig. 3b). However, pathological examination confirmed that it was still adenocarcinoma, indicating that small-cell lung cancer transformation did not occur in this case. Next generation sequencing (1021 Gene panel from Geneplus China Corp.) of the left axillary lymph node tissue after failure of osimertinib therapy found eight mutated genes (EGFR L858R44.6%, TP53 40.0%, ABL2 29.1%, FAT1 27.3%, NF2 16.2%, CSMD3 8.9%, RET 8.9%, OR6F1 1.0%). But only TP53 mutation seems significant. We could not find co-existing mutations like PI3K-AKT pathway. Matteo Canale [4] reported that the risk of disease progression was three times higher in patients with TP53 mutations than in those without TP53 mutation. Thus we wonder if TP53 nutation is another possible mechanism of this case.

What is more, we found the abundance of T790 M and L858R mutation dropped from 11.7 to 0.00% and from 49.8 to 34.5% after taking osimertinib, respectively, while the disease continuously progressed. Thus, the result showed that there was no relation between the decrease of EGFR mutation abundance and tumor response. Zhang, B.O [5] found ddPCR was more sensitive in detecting EGFR mutation, especially for low abundance. In this case, the patient was diagnosed with EGFR T790 M-negative mutation using ARMS-qPCR, but retrospective detection using ddPCR found that EGFR T790 M mutation was positive (0.25%). So, if low abundance of EGFR mutation is observed, ddPCR is recommended.

Our clinical trial proves it’s a good strategy to perform concurrent local radiotherapy for first-line EGFR-TKI and local progression in advanced NSCLC patients [6]. In this case, the patient received concurrent erlotinib with local radiotherapy as first-line therapeutic strategy and the PFS was14 months. However, primary resistance to osimertinib was observed in this patient. Thus, we wonder if radiotherapy plays a role in primary resistance to osimertinib. Hirata H and his colleagues [7] reported that acquired resistance to TKIs appears to be associated with low efficacy of radiotherapy, but there are no reports about the relationship between primary resistance to osimertinib and radiotherapy. In conclusion, this is a case report of primary resistance to osimertinib in erlotinib-pretreated lung adenocarcinoma with EGFR T790 M mutation. The mechanism is unclear. T790 M mutation disappearance, TP53 mutation and radiotherapy could be associated.

## Abbreviations

ddPCR: Droplet digital polymerase chain reaction
EGFR-TKI: Epidermal growth factor receptor tyrosine kinase inhibitor
NGS: Next generation sequencing
NSCLC: Non-small cell lung cancer
NSE: Neuron specific enolase
